# Supplementary material for: Policy formulation and actor roles in the expanded Kenyan free maternity policy (Linda Mama): A policy analysis
Source: PLOS Glob Public Health. 2024 Nov 21;4(11):e0002796. doi: 10.1371/journal.pgph.0002796 (PMC11581349; doi:10.1371/journal.pgph.0002796)
Supplement: S1 Appendix — (PDF) [file pgph.0002796.s002.pdf]

Appendix 1: Semi-structured interview guide with Ministry of Health officials, National Health Insurance Funds officials, donors, civil society including representatives of health service providers

Linda Mama Process evaluation - Data Collection Tools

Tool 1: Semi-structured interview guide: in - depth interviews with national (MOH and NHIF officials), other health financing stakeholders (e.g. KHF, Private hospital associations, CHAK, Development partners)

Formal title: A process evaluation of the implementation of Linda Mama Free Maternity Program in selected counties in Kenya.

Lay Title: Assessing the implementation of the Linda Mama Program in selected counties in Kenya.

Interviewee profile

1. Could you introduce yourself, your organization and your organization mandate.
2. Could you tell us a little bit about your role in this organization and how long you have been in this role.

Emergence of the Linda Mama program

1. Could you explain to us what problems/challenges were identified with the previous free maternity services?
2. What were the proposed solutions at the time?
3. What were the debates surrounding potential solutions to the challenges experienced by the previous free maternity services?
4. Why was the Linda Mama program the preferred choice among other possible options?
5. Who were the stakeholders actively involved in the formulation of the Linda mama program?
6. What was the role of these stakeholders in the formulation process?
7. Could you describe the status of stakeholder support for the transition from the previous free maternity services to Linda Mama? Who were in support and why? Who were in opposition and why?

Experience and Fidelity of the Linda mama implementation

8. Is there a policy document that outlines the Linda Mama program?
9. Could you describe the organizational arrangement of the Linda Mama program (who does what?)

Funding flows

10. What are the sources of funds for the Linda Mama Program?
11. Are the funds allocated for the Linda Mama program considered sufficient? Why?
12. According to the Linda mama policy, how should these funds flow from the source to healthcare facilities?
13. Is this how the funds flow in practice? Why?
14. What is your experience in terms of challenges and strengths of the funds flow arrangements?

Benefit Package and Access

15. What services are Linda Mama program beneficiaries formally entitled to?
16. Do Linda Mama beneficiaries receive all these benefits in practice? Why?
17. Who are the intended beneficiaries of the Linda Mama program benefits?
18. Who accesses the Linda Mama program benefits in practice?

19. Who has challenges accessing Linda Mama services? Are there any initiatives to ensure that there is extended reach to them?
20. What are the potential challenges for the poor, adolescents, and those lacking education in accessing Linda Mama services?
21. How does Linda Mama influence access to services?
22. How does Linda Mama influence quality of care?
23. What are the potential barriers that inhibit beneficiaries from accessing their entitlements under the Linda mama program?
24. What are the potential facilitators that enhance beneficiaries' access to their entitlements under the Linda mama program?

Strategic Purchasing

25. In what healthcare facilities can Linda Mama program beneficiaries access Linda Mama services? (probe for different facility levels and ownership)
26. How are these healthcare facilities selected to provide healthcare services under the Linda Mama program in practice?
27. Do Linda Mama beneficiaries access Linda Mama services from all these facility options in practice? Why? (probe for different facility levels and ownership)
28. According to Linda Mama policy, how and on what basis should healthcare providers be paid for providing services for Linda Mama beneficiaries?
29. Is this how healthcare providers are paid in practice? Why?
30. What are the challenges and strengths of the provider payment arrangements under the Linda Mama program in relation to the facility functioning and provider behavior?
31. What is the formal claims procedure for healthcare facilities under the Linda Mama program?
32. Is this how the claims process is done in practice? Why?
33. In practice, who reviews the claims from the health facilities? How are the claims verified? What happens if the claims are not approved?
34. What are the challenges and strengths of the claims procedures under the program?
35. What are the payment disbursement arrangements to healthcare facilities under the Program? (probe frequency)
36. What are the challenges and strengths of the payment disbursement arrangement s? Why? (probe delays, unpredictability in timing and amount)
37. Do healthcare facilities have the freedom to spend funds from the program according to their priorities? Why?
38. How does this (freedom or lack of it) affect service delivery under the program?
39. What reporting requirements exist between the NHIF and the ministry of health for the Linda Mama program?
40. How well do these reporting requirements work in practice? Why?
41. How does the state of the reporting requirement affect the implementation of the Linda Mama Program?
42. What mechanisms are in place for the NHIF to ensure that healthcare facilities offer services that are of good quality under the Linda Mama Program?
43. How well do these quality assurance mechanisms work? Why?

Future of the program

44. How might the Linda Mama Program, and how it currently functions, inform the development and implementation of the UHC pilots?
45. What improvements to Linda Mama should be made or prioritized to increase access to care, improve quality of care and improve the functioning of the program?

Conclusion: Thank him/her for their participation and conclude the discussion.

Appendix 2: Semi-structured interview guide with County and sub-county officials

Research title: "The Kenyan "Free" Maternal Health Policy: Lessons for Universal Health Coverage on Cost and Quality of Care – A Case Study Approach"

**Data of discussion:**	**Interviewer:**
**Venue:**	**Note taker:**
**Start time:**	**Stop time:**
**Interviewee's code:**	
**Interview completed:**	**Yes** ☐ **No** ☐
**Reason for incomplete interview**	
**Interviewers' remarks about the interview (debrief)**<br>**1.**<br>**2.**<br>**3.**<br>**4.**<br>**5.**	

**Questions**
**Introduction and background**<br>**Key informant profile**<br>1. Could you introduce yourself and your organization<br>2. Could you tell us a little bit about your role and how long you have performed it
**Policy streams: Goals and the objectives of the policy (content)**<br>3. In your opinion, what was the goal and the objective of the Linda mama policy? Has it achieved that objective?<br>4. Were you involved in the design? **Probe:** how did you get to hear about the policy?<br>5. What are some of the services covered in the policy?<br>6. What are some of the quality, cost, and coverage guidelines that were put in place at the design? How are they adhered to? Are there policy documents to detail the guidelines?<br><br>**Context**<br>***Situational factors***<br><br>7. In your opinion, was there any particular situation or activity or research that brought about changes in how linda mama policy is implemented in this county? **Probe:** what situation or activity or research was it? How did it influence formulation of the policy?<br>***Socio-political factors***<br>8. Were there county government political events or influencers of or against the Linda Mama programme as a policy change? ***Probe:*** which ones and how did they influence?<br>9. Are there other similar programs that are limiting the achievement of the policy or supporting it? How do you manage the two?
**process stream: Causal issues and technical aspects**<br>10. Are you receiving payments under the Linda mama policy? How does the process take place?<br>11. What are some of the services that are free? Is every pregnant mother exempted? If not, why?

12. What is the payment tariff for the services rendered? E.g. ANC? Normal deliveries? CS? Assisted delivery? PNC? Other? Complications? ***Probe:*** whether the tariffs are fixed or whether they vary by case?
13. Is Linda mama policy working effectively now as it was communicated to you? (If not, why not?)
14. Are the funds you are receiving adequate? How do they calculate the amount? Is there any limit on numbers of delivery patients that you can get reimburse for?
15. How are the funds allocated to the hospitals?
16. What are the mechanisms for paying the funds? How well do they operate? ***Probe:*** the funds of the implementation of the policy arrived at the county level? Do you think it is sustainable?
17. Do mothers make payments for anything (supplies, materials, etc.)? How much do they contribute if at all they do?
18. In your opinion do you think the policy has affected the quality of services? ***Probe:*** some of the quality issues that have been affected? Have they been affected positively or negatively?
19. How do you monitor the achievement of the policy?

Resource stream: Human resource (*Actors*)- (power, staff time, ownership and reputation)

20. In your opinions who are the key players at a county level involved in the implementation of the policy? ***Probe:*** how do they that ensure the policy is implemented as planned? What are their roles?
21. What is the relationship between the actors you have mentioned?
22. In your opinion what is the influence of the actors? Who makes the decisions and how do they influence the implementation of the policy?
23. Are there changes in the number of staff since the policy began? **Probe:** is it a positive change or a negative change?
24. In your opinion, what is the difference in the amount of work done by the staff and are they motivated to conduct the work? Why yes/no?
25. In your opinion, how do you perceive the quality outcomes of the policy
26. Has the policy changed the level of quality in your facility? What elements of quality? Have they changed for the better or for the worse?
27. What are some of the changes that you have put in place as a result of the new policy? **Probe:** Investments in structure of the facility, motivation of staff etc.
28. Do you charge the patients something additional other than what is stipulated in the policy? How do you deal with the very poor patients who are not able to afford transport to the facility?

financial resources

29. How do the reimbursed moneys reach the facilities and in what areas do you spend it? Before spending, what are the processes involved?
30. In your opinion, is the cost of reimbursement of the services adequate?

Other opinions

31. What are some of the challenges you have experienced with the Linda mama policy in your facility?
32. Are you happy with the way it is being handled? Why yes/no?
33. How can we improve the operations of the policy?

Appendix 3: Semi-structured interview guide with facility in-charges and health-care providers

Research title: "The Kenyan "Free" Maternal Health Policy: Lessons for Universal Health Coverage on Cost and Quality of Care – A Case Study Approach"

**Data of discussion:**	**Interviewer:**
**Venue:**	**Note taker:**
**Start time:**	**Stop time:**
**Interviewee's code:**	
**Interview completed:**	**Yes** ☐ **No** ☐
**Reason for incomplete interview**	
**Interviewers' remarks about the interview (debrief)**<br>**1.**<br>**2.**<br>**3.**<br>**4.**<br>**5.**	

**Questions**
**Introduction and background**<br>**Key informant profile**<br>1. Could you introduce yourself and your organization<br>2. Could you tell us a little bit about your role and how long you have performed it
**Policy streams**<br>**Goals and the objectives of the policy**<br>3. In your opinion, what was the goal and the objective of the Linda mama policy? Has it achieved that objective?<br>4. What are some of the services covered in the policy?<br>5. What are some of the quality guidelines that were put in place at the design? How are they adhered to? Are there policy documents to detail the quality guidelines?<br><br>**Quality**<br><br>6. In your opinion, was there any particular situation or activity or research that brought about changes in how Linda mama policy is implemented in this county? Probe: what situation or activity or research was it? How did it influence formulation of the policy?<br>***Socio-political factors***<br>7. Were there county government political events or influencers of or against the Linda Mama programme as a policy change? ***Probe:*** which ones and how did they influence?<br>8. Are there other similar programs that are limiting the achievement of the policy or supporting it? How do you manage the two?<br><br>**Cost**<br>9.
**Process stream: Causal issues and technical aspects**<br>10. Are you receiving payments under the Linda mama policy? How does the process take place?

11. What are some of the services that are free? Is every pregnant mothers exempted? If not, why?
12. What is the payment tariff for the services rendered? E.g. ANC? Normal deliveries? CS? Assisted delivery? PNC? Other? Complications? ***Probe:*** whether the tariffs are fixed or whether they vary by case
13. Is Linda mama policy working effectively now at it was communicated to you? (If not, why not?)
14. Are the funds you are receiving adequate? How do they calculate the amount? Is there any limit on numbers of delivery patients that you can get reimburse for?
15. How are the funds allocated to the hospitals?
16. What are the mechanisms for paying the funds? How well do they operate? ***Probe:*** the funds of the implementation of the policy arrived at the county level
17. Do mothers make payments for anything (supplies, materials, etc.)? How much do they contribute if at all they do?
18. In your opinion do you think the policy has affected the quality of services? ***Probe:*** some of the quality issues that have been affected? Have they been affected positively or negatively?
19. How do you monitor the achievement of the policy?

Resource stream: Human resource (*Actors*)- (power, staff time, ownership and reputation)

20. In your opinions who are the key players at a county level involved in the implementation of the policy? ***Probe:*** how do they that ensure the policy is implemented as planned? What is their roles?
21. What is the relationship between the actors you have mentioned?
22. In your opinion what is the influence of the actors? Who makes the decisions and how do they influence the implementation of the policy?
23. Are there changes in the number of staff since the policy began? Probe: is it a positive change or a negative change?
24. In your opinion, what is the difference in the amount of work done by the staff and are they motivated to conduct the work? Why yes/no?
25. In your opinion, how do you perceive the quality outcomes of the policy
26. Has the policy changed the level of quality in your facility? What elements of quality? Have they changed for the better or for the worse?
27. In your opinion, is the cost of reimbursement of the services adequate?
28. What are some of the changes that you have put in place as a result of the new policy? ***Probe:*** Investments in structure of the facility, motivation of staff etc.
29. Do you charge the patients something additional other than what is stipulated in the policy? How do you deal with the very poor patients who are not able to afford transport to the facility?
30. How do the reimbursed moneys reach the facilities and in what areas do you spend it? Before spending, what are the processes involved?

Other opinions

31. What are some of the challenges you have experienced with the Linda mama policy in your facility?
32. Are you happy with the way it is being handled? Why yes/no?
33. How can we improve the operations of the policy?
