# Supplementary material for: Policy formulation and actor roles in the expanded Kenyan free maternity policy (Linda Mama): A policy analysis
Source: PLOS Glob Public Health. 2024 Nov 21;4(11):e0002796. doi: 10.1371/journal.pgph.0002796 (PMC11581349; doi:10.1371/journal.pgph.0002796)
Supplement: Attachment — Submitted filename: PLOS- The policy formulation process.docx [file pgph.0002796.s003.docx]

**Summary:** This study was based on the topic “The policy formulation process, and the role of actors in the policy formulation and implementation process: A policy analysis of the Kenyan free maternity policy”. The subject matter is very important, particularly for low-and-middle-income countries. 

There are major and minor revisions present in the current submission that need to be addressed. Since this is a very important topic, I would like to strongly encourage the authors to consider addressing the issues raised to strengthen the work for reconsideration.

**Abstract**

Major Compulsory Revisions
Overall: The abstract in its current form needs more strengthening. The results should state clearly the contextual factors that led to the policy formulation. What policy processes were adopted. Are there any identified gaps in the policy content. Are there some powerful actors who were excluded but who you think would have made the policy better if they were included? Did the actors’ practices of power influence the policy development and implementation positively or negatively? So basically, what lessons can be learnt from the formulation and implementation of ‘Linda Mama” policy

Line 42: The authors mentioned they conducted an exploratory qualitative study which involved document review, key informant interviews (KIIs) with national stakeholders, and in-depth interviews. They however did not state the study design used. This needs to be provided

Line 44-46: The authors stated that the used a theoretical framework capturing the preliminary situation analysis of the policy, the processes, the content, and the stakeholders' roles in the formulation and implementation. It would be useful to state the specific framework that was used upfront to help the reader. Also, theoretical framework wasn’t mentioned in the manuscript but conceptual and analytic framework. These are different from theoretical framework. This has to be reconciled.

Line 50: SDGs should be written in full first. Also, the sentence ‘…to improve the quality of maternal and neonatal care and eliminate financial barriers’ seems incomplete. Eliminate financial barriers to what?

**Body of Manuscript**

**Introduction**

Minor Essential Revisions
Line 75: ‘due inadequate’ should be ‘due to inadequate’.

Line 78-79: the sentence “It provided a package of essential health services for pregnant women accessed by all in the targeted population based on need and not the ability to pay” is not clear

Line 85: the authors should consider replacing “researchers” which runs through the rest of the manuscript with “studies”.

Line 88: The word policy should be deleted since it’s more like a repetition from line 86.

**Methods**
Major Compulsory Revisions

*The guiding conceptual and analytical framework*

The health policy analysis triangle framework by Walt and Gilson is appropriate and adequate enough for this study. The framework captures the context, processes, content and actors involved in the formulation as well as the implementation of the policy and thus helps to better understand the health policy reform and to plan for effective implementation. I therefore think the use of Hercot et al and SHIELD project which are not to support the framework but as separate frameworks/applied approaches are redundant and should be deleted

The authors should briefly describe the health policy analysis triangle framework and state what constitutes the context, process, content and actor components based on this study and how they applied it in this study. What were assessed or examined under each component?

The statement “It captures the background of the policy, derived from Hercot et al.’s [25] work, which focuses on the preliminary situation analysis and setting the priorities of the policy (an understanding of the origin of the policy). It also emphasizes the portrayal of an existing window of opportunity needed to restrict the ‘inventory phase’ of a policy” is captured by the context of the policy process under the health policy analysis triangle framework when the framework is applied well and therefore should be deleted

The use of preliminary situation analysis should be replaced with context of the policy development

The statement “(whose roles, power, and influence during formulation and implementation was analysed through a stakeholder’s analysis [28-30])” should be deleted

*Study design*

Line 137: the authors should state the specific study design used, e.g. case study, cross-sectional etc

*Study population and sampling*

Line 152: FMP should be written in full first

Line 153-154: National health insurance agency does not correspond to NHIF

*Data collection and analysis*

The study lacks detailed description of the data collection procedure. Which data were collected under the context, process, content and actor components of the framework? What information were obtained from the informant categories. How was the data collected for the document review or what instruments were used. Which aspects of the policy development and implementation were covered by the instruments?

Line 167: BO needs to be defined

**Results**
Major Compulsory Revisions

This section requires reformatting. This is essential because the current format is very difficult to make sense of as the manner of presentation is difficult for the reader to make sense of the value to the research findings.

In particular, the presentation of headings/themes without sub-headings or subthemes. This makes it very difficult for the reader to appreciate the value of the research findings. I will recommend that the authors present the findings under five headings/themes: 1) context of FM policy formulation 2) the FM policy formulation processes 3) content of the FM policy 4) Influence of actors over FM policy formulation and 5) influence of actors over FM policy implementation. Then under each, have sub-themes based on inductive analysis instead of the current format.

Line 189-190: Authors should replace the heading with ‘context of FM policy formulation’

Line 193: FM should be written in full first

Line 195-196: Instead of bolding, a sub-heading/theme should be created.

Line 198: “the Vision 2030” of what?

Line 200:” with” consider delete

Line 208: a subtheme/heading is needed

Line 209: Which of the SDGs? There is the need to be specific

Line 218-219: a sub-theme/heading needed

Line 234: a subtheme/heading needed

Line 234-236: the sentence Some respondents perceived the FMS policy to be a political tool used by the government to fulfil a campaign agenda, as captured in the president’s Jubilee Party 2013 Presidential campaign Manifesto” is a bit confusing. The authors should make it clear whether it is the Linda Mama policy they evaluated or previous FM policy in 2013

Lines 236-237: The sentence “some of the respondents highlighted that the goal of the policy was to fulfil part of the Big Four agenda that the then…”. What are the Big Four agenda?

Line 246: a subtheme/heading needed

Lines 261-262: a subtheme/heading needed

Line 277-278: a subtheme/heading needed

Line 287: a subtheme/heading needed

Lines 290-291: Repetition of process evaluation. Consider delete

Line 292: what is inflated utilization number

Line 305: Authors should consider making the heading “the FM policy formulation processes’

Line 306: a subtheme/heading needed

Line 342: a subtheme/heading needed

Line 353 and 355: what are quantiles 1,2 and 3

Line 376: a subtheme/heading needed

Line 397: a subtheme/heading needed

Line 430-431: a subtheme/heading needed

Line 457: a subtheme/heading needed

Line 481-482: consider breaking this into two main headings: 1) Influence of actors over FM policy formulation and 2) influence of actors over FM policy implementation. What are the bottlenecks or successes identified?

Line 483: a subtheme/heading needed

Line 488: a subtheme/heading needed

Line 522: a subtheme/heading needed

Line 531: a subtheme/heading needed. Also, the use of “joined-up government at the centre”, what does it mean?

Line 590: “important role” in what?

Line 600: a subtheme/heading needed

**Discussions**

Major Essential Revisions

Overall, the discussion needs a major revision. The discussion should focus on the successes, failures/bottlenecks/constraints or the lessons learnt from the policy development and implementation. That is, it should speak to the findings on the contextual factors that led to the policy formulation, the policy processes adopted, any identified gaps in the policy content and the influence of the actors in formulation and implementation in the context of current literature.

Line 657: what window of opportunity did you find in this study

Line 662-663: What does the statement “Further, by building on the lessons from the previous FM policy, the policy is taking the ebbs and flows fluid process instead of remaining in a fixed static form” mean?

Line 668-669: What does “Sitting at the centre of the policy triangle is the issue of power, and its role in decision-making is incontrovertible” mean?

Line 677: “(such as the developed concept of social franchise networks)”. What does it mean and where is it coming from?

Line 683-685: “The NGOs representing the interests of the private sector in the informal settlements devised methods to bypass the political process at the formulation to engage the sector and shape the price debate at the grassroots level rather than at the top”. Is this found in the study?

Line 711: Faith Based Organisations should be written in abbreviated form

Line 712: Nongovernmental Organisations should be written in abbreviated form

Line 726: The use of “stakeholders” should be replaced with “actors”, its more appropriate. This should reflect in the relevant sections of the manuscript”

Line 750-751: This sentence “The finding shows that the beneficiaries’ representatives were significant because they were classified in the FM reform as the latter” is not clear

Line 766: What does PS mean

**Conclusion**

The conclusion should speak **to** the successes, bottlenecks/constraints or the lessons learnt from the policy development and implementation that can offer support for policy reform. That is, it should speak to the contextual factors that led to the policy formulation, the policy processes adopted, any identified gaps in the policy content and the influence of the actors in formulation and implementation.
