# Supplementary material for: Policy formulation and actor roles in the expanded Kenyan free maternity policy (Linda Mama): A policy analysis
Source: PLOS Glob Public Health. 2024 Nov 21;4(11):e0002796. doi: 10.1371/journal.pgph.0002796 (PMC11581349; doi:10.1371/journal.pgph.0002796)
Supplement: Attachment — Submitted filename: Point by point response to the reviewers.docx [file pgph.0002796.s004.docx]

**Point by point response to the reviewers**

**Journal Requirements:**

1. Please include a complete copy of PLOS’ questionnaire on inclusivity in global research in your revised manuscript. Our policy for research in this area aims to improve transparency in the reporting of research performed outside of researchers’ own country or community. The policy applies to researchers who have travelled to a different country to conduct research, research with Indigenous populations or their lands, and research on cultural artefacts. The questionnaire can also be requested at the journal’s discretion for any other submissions, even if these conditions are not met. Please find more information on the policy and a link to download a blank copy of the questionnaire here: <https://journals.plos.org/globalpublichealth/s/best-practices-in-research-reporting>. Please upload a completed version of your questionnaire as Supporting Information when you resubmit your manuscript.

We have now provided a completed version of your questionnaire as Supporting Information

2. Please provide separate figure files in .tif or .eps format only and remove any figures embedded in your manuscript file. Please also ensure all files are under our size limit of 10MB.

For more information about figure files please see our guidelines:

<https://journals.plos.org/globalpublichealth/s/figures>

<https://journals.plos.org/globalpublichealth/s/figures#loc-file-requirement>

We have separated the figures and ensured they meet the guidelines as stated.

3. We have noticed that you have uploaded Supporting Information files, but you have not included a list of legends. Please add a full list of legends for your Supporting Information files after the references list.

We have provided a full list of legends for the Supporting Information files after the references list.

**Additional Editor Comments (if provided):**

**Reviewers' comments:**

**5. Review Comments to the Author**

Please use the space provided to explain your answers to the questions above. You may also include additional comments for the author, including concerns about dual publication, research ethics, or publication ethics. (Please upload your review as an attachment if it exceeds 20,000 characters)

**Reviewer #1:**

**Summary:** This study was based on the topic “The policy formulation process, and the role of actors in the policy formulation and implementation process: A policy analysis of the Kenyan free maternity policy”. The subject is very important, particularly for low-and-middle-income countries.

We thank the reviewer for this positive comment.

There are major and minor revisions present in the current submission that need to be addressed. Since this is a very important topic, I would like to strongly encourage the authors to consider addressing the issues raised to strengthen the work for reconsideration.

**Abstract**

**Major Compulsory Revisions**

Overall: The abstract in its current form needs more strengthening. The results should state clearly the contextual factors that led to the policy formulation. What policy processes were adopted. Are there any identified gaps in the policy content. Are there some powerful actors who were excluded but who you think would have made the policy better if they were included? Did the actors’ practices of power influence the policy development and implementation positively or negatively? So basically, what lessons can be learnt from the formulation and implementation of ‘Linda Mama” policy

We have now rewritten the abstract to make it flow better and capture elements as suggested by reviewers.

Line 42: The authors mentioned they conducted an exploratory qualitative study which involved document review, key informant interviews (KIIs) with national stakeholders, and in-depth interviews. They however did not state the study design used. This needs to be provided

We have now rewritten the abstract to make it flow better and capture elements as suggested by reviewers.

Line 44-46: The authors stated that the used a theoretical framework capturing the preliminary situation analysis of the policy, the processes, the content, and the stakeholders' roles in the formulation and implementation. It would be useful to state the specific framework that was used upfront to help the reader. Also, theoretical framework wasn’t mentioned in the manuscript but conceptual and analytic framework. These are different from theoretical framework. This has to be reconciled.

We have now rewritten the abstract to make it flow better and capture elements as suggested by reviewers.

Line 50: SDGs should be written in full first. Also, the sentence ‘…to improve the quality of maternal and neonatal care and eliminate financial barriers’ seems incomplete. Eliminate financial barriers to what?

We have now rewritten the abstract to make it flow better and capture elements as suggested by reviewers.

**Body of Manuscript**

**Introduction**

**Minor Essential Revisions**

Line 75: ‘due inadequate’ should be ‘due to inadequate’.

We have now made this correction.

Line 78-79: the sentence “It provided a package of essential health services for pregnant women accessed by all in the targeted population based on need and not the ability to pay” is not clear: We have re-written the sentence and made it clearer in line with the flow of the section.

Line 85: the authors should consider replacing “researchers” which runs through the rest of the manuscript with “studies”.

We have now made this correction as suggested.

Line 88: The word policy should be deleted since it’s more like a repetition from line 86.

We have now made this correction as suggested.

**Methods**

**Major Compulsory Revisions**

**The guiding conceptual and analytical framework**

The health policy analysis triangle framework by Walt and Gilson is appropriate and adequate enough for this study. The framework captures the context, processes, content and actors involved in the formulation as well as the implementation of the policy and thus helps to better understand the health policy reform and to plan for effective implementation. I therefore think the use of Hercot et al and SHIELD project which are not to support the framework but as separate frameworks/applied approaches are redundant and should be deleted.

We thank the reviewer for the suggestions. However, we feel that just using the health policy analysis triangle framework, as captured by Walt and Gilson, leaves out key elements that our study brings out. For example, the Policy Triangle consists of four elements: context (why need this policy), content (what is the policy mainly about), process (how was the policy brought forward and implemented) and actors (who participates in and influences formulation and implementation of the policy) which we have highlighted adequately in the context. However, it does not capture the actual origin of the policy, which is based on the actual priorities (and how the priorities that led to the policy were brought about). Further, it doesn’t adequately capture the window of opportunity of how these elements came to place and which are key to setting the priorities. Instead, we have built a conceptual framework drawn from other authors to capture what we needed to highlight based on our study objective, which is what we have presented. We feel that the use of only policy analysis triangle by Walt and Gilson is limiting.

As such, rather than just have one framework, we created a guiding conceptual framework derived from reviews and strengths of several components and alighned into a focused way for our work.

Therefore, we have rewritten this section to enhance clarity as raised by the reviewer, making it clearer.

The authors should briefly describe the health policy analysis triangle framework and state what constitutes the context, process, content and actor components based on this study and how they applied it in this study. What were assessed or examined under each component?

We thank the reviewer for the suggestions for the suggestion. We have rewritten this sections reflecting on what each of the component covers.

The statement “It captures the background of the policy, derived from Hercot et al.’s [25] work, which focuses on the preliminary situation analysis and setting the priorities of the policy (an understanding of the origin of the policy). It also emphasizes the portrayal of an existing window of opportunity needed to restrict the ‘inventory phase’ of a policy” is captured by the context of the policy process under the health policy analysis triangle framework when the framework is applied well and therefore should be deleted

We thank the reviewer for the suggestions. We have rewritten this section to enhance the clarity of each of the component covers but also rewritten this element that the reviewer has captures without losing the meaning of what our work is about.

The use of preliminary situation analysis should be replaced with context of the policy development

We thank the reviewer for the suggestions for the suggestion. We have rewritten this section into ‘background priorities and the context of the policy.’ This allows us not to lose the elements of the background priorities that do not exist in the context section of the health policy triangle.

The statement “(whose roles, power, and influence during formulation and implementation was analysed through a stakeholder’s analysis [28-30])” should be deleted

We thank the reviewer for the suggestions. We have rewritten this section to enhance the clarity of each of the element our study is communicating.

**Study design**

Line 137: the authors should state the specific study design used, e.g. case study, cross-sectional etc

We thank the reviewer for this comment. We have now made this correction as suggested.

**Study population and sampling**

Line 152: FMP should be written in full first:

We have included in full the words that make FMP in the introduction section.

Line 153-154: National health insurance agency does not correspond to NHIF

We have included in full the words that make NHIF in the introduction section.

**Data collection and analysis**

The study lacks detailed description of the data collection procedure. Which data were collected under the context, process, content and actor components of the framework? What information were obtained from the informant categories. How was the data collected for the document review or what instruments were used. Which aspects of the policy development and implementation were covered by the instruments?

We thank the reviewer for this comment. We have now made detailed description of the data collection procedure.

Line 167: BO needs to be defined

We have now defined BO.

**Results**

**Major Compulsory Revisions**

This section requires reformatting. This is essential because the current format is very difficult to make sense of as the manner of presentation is difficult for the reader to make sense of the value to the research findings. In particular, the presentation of headings/themes without sub-headings or subthemes. This makes it very difficult for the reader to appreciate the value of the research findings. I will recommend that the authors present the findings under five headings/themes: 1) context of FM policy formulation 2) the FM policy formulation processes 3) content of the FM policy 4) Influence of actors over FM policy formulation and 5) influence of actors over FM policy implementation. Then under each, have sub-themes based on inductive analysis instead of the current format.

We thank the reviewers for this comment and suggestion. To align with the work that we aimed to achieve from the onset, we have presented the Background and context of the policy; Policy formulation processes; Content of the policy and the Roles of the stakeholders in the formulation and the implementation.

Line 189-190: Authors should replace the heading with ‘context of FM policy formulation’

We thank the reviewers for this comment. We have now rewritten the section to capture background priorities and the context of the policy which defined the policy's aims while considering political, social, and economic factors at both local and national levels.

Line 193: FM should be written in full first

We thank the reviewers for this comment. We have now correct eths throughout the document

Line 195-196: Instead of bolding, a sub-heading/theme should be created. We thank the reviewers for this comment. We have now created subheadings and themes throughout the document.

Line 198: “the Vision 2030” of what? We have now corrected this part to Kenya Vision 2030 framework.

Line 200:” with” consider delete. This has now been corrected

Line 208: a subtheme/heading is needed. We thank the reviewers for this comment. We have now created subheadings and themes throughout the document.

Line 209: Which of the SDGs? There is the need to be specific. We have now included the specificity of the SDGs. SDGs 3.1, 3.2, and 3.7, which focus on reducing maternal mortality, ending preventable deaths of newborns and children under 5, and ensuring universal access to sexual and reproductive health-care services.

Line 218-219: a sub-theme/heading needed We thank the reviewers for this comment. We have now created subheadings and themes throughout the document.

Line 234: a subtheme/heading needed: We thank the reviewers for this comment. We have now created subheadings and themes throughout the document.

Line 234-236: the sentence Some respondents perceived the FMS policy to be a political tool used by the government to fulfil a campaign agenda, as captured in the president’s Jubilee Party 2013 Presidential campaign Manifesto” is a bit confusing. The authors should make it clear whether it is the Linda Mama policy they evaluated or previous FM policy in 2013: We thank the reviewers for this comment. We have now corrected this to reflect that the policy is about the expanded FMP.

Lines 236-237: The sentence “some of the respondents highlighted that the goal of the policy was to fulfil part of the Big Four agenda that the then…”. What are the Big Four agenda? In the same sentence, we have now highlighted what the big four agendas are.

Line 246: a subtheme/heading needed. We thank the reviewers for this comment. We have now created subheadings and themes throughout the document.

Lines 261-262: a subtheme/heading needed. We thank the reviewers for this comment. We have now created subheadings and themes throughout the document.

Line 277-278: a subtheme/heading needed. We thank the reviewers for this comment. We have now created subheadings and themes throughout the document.

Line 287: a subtheme/heading needed. We thank the reviewers for this comment. We have now created subheadings and themes throughout the document.

Lines 290-291: Repetition of process evaluation. Consider delete. We thank the reviewer for this comment. We have now corrected the section.

Line 292: what is inflated utilization number. We have corrected this to mean Overstated utilization figures

Line 305: Authors should consider making the heading “the FM policy formulation processes’ We have now corrected this as suggested by the reviewer.

Line 306: a subtheme/heading needed. We thank the reviewers for this comment. We have now created subheadings and themes throughout the document.

Line 342: a subtheme/heading needed. We thank the reviewers for this comment. We have now created subheadings and themes throughout the document.

Line 353 and 355: what are quantiles 1,2 and 3. We have now corrected this from quantiles to quintiles and defined what they are and cited a reference to the work.

Line 376: a subtheme/heading needed. We thank the reviewers for this comment. We have now created subheadings and themes throughout the document.

Line 397: a subtheme/heading needed. We thank the reviewers for this comment. We have now created subheadings and themes throughout the document.

Line 430-431: a subtheme/heading needed. We thank the reviewers for this comment. We have now created subheadings and themes throughout the document.

Line 457: a subtheme/heading needed. We thank the reviewers for this comment. We have now created subheadings and themes throughout the document.

Line 481-482: consider breaking this into two main headings: 1) Influence of actors over FM policy formulation and 2) influence of actors over FM policy implementation. What are the bottlenecks or successes identified? We have made the suggested correction in this line

Line 483: a subtheme/heading needed. We thank the reviewers for this comment. We have now created subheadings and themes throughout the document.

Line 488: a subtheme/heading needed. We thank the reviewers for this comment. We have now created subheadings and themes throughout the document.

Line 522: a subtheme/heading needed. We thank the reviewers for this comment. We have now created subheadings and themes throughout the document.

Line 531: a subtheme/heading needed. Also, the use of “joined-up government at the centre”, what does it mean? We have now clarified this to mean Central (national) government coordination in the arrangement.

Line 590: “important role” in what? We have now clarified this to ‘important advisory role’

Line 600: a subtheme/heading needed. We thank the reviewers for this comment. We have now created subheadings and themes throughout the document.

**Discussions**

**Major Essential Revisions**

Overall, the discussion needs a major revision. The discussion should focus on the successes, failures/bottlenecks/constraints or the lessons learnt from the policy development and implementation. That is, it should speak to the findings on the contextual factors that led to the policy formulation, the policy processes adopted, any identified gaps in the policy content and the influence of the actors in formulation and implementation in the context of current literature.

We have rewritten the whole discussion section to align with the reviewer’s suggestions bringing out the key elements as per the objectives.

Line 657: what window of opportunity did you find in this study. We have now rewritten the discussion section (paragraph one) to show how the window of opportunity came into place.

Line 662-663: What does the statement “Further, by building on the lessons from the previous FM policy, the policy is taking the ebbs and flows fluid process instead of remaining in a fixed static form” mean? We have now rewritten the discussion section (paragraph one) to clarify the meaning of the policy is taking the ebbs and flows fluid process instead of remaining in a fixed static form.

Line 668-669: What does “Sitting at the centre of the policy triangle is the issue of power, and its role in decision-making is incontrovertible” mean? We have now rewritten the discussion section (paragraph one) to clarify the meaning of *‘Sitting at the centre of the policy triangle is the issue of power, and its role in decision-making is incontrovertible’.*

Line 677: “(such as the developed concept of social franchise networks)”. What does it mean and where is it coming from? We have now removed this section to avoid introducing new subjects.

Line 683-685: “The NGOs representing the interests of the private sector in the informal settlements devised methods to bypass the political process at the formulation to engage the sector and shape the price debate at the grassroots level rather than at the top”. Is this found in the study? Yes, this is found under the section on Private sector's key role in policy formulation.

Line 711: Faith Based Organisations should be written in abbreviated form We have now made this correction

Line 712: Nongovernmental Organisations should be written in abbreviated form We have now made this correction

Line 726: The use of “stakeholders” should be replaced with “actors”, its more appropriate. This should reflect in the relevant sections of the manuscript” We have now made the corrections as suggested by the review throughout the document.

Line 750-751: This sentence “The finding shows that the beneficiaries’ representatives were significant because they were classified in the FM reform as the latter” is not clear We have now clarified the meaning of this statement.

Line 766: What does PS mean We included in the result section the full wording for PS to mean principal secretary

**Conclusion**

The conclusion should speak to the successes, bottlenecks/constraints or the lessons learnt from the policy development and implementation that can offer support for policy reform. That is, it should speak to the contextual factors that led to the policy formulation, the policy processes adopted, any identified gaps in the policy content and the influence of the actors in formulation and implementation. We have now rewritten the conclusion section to capture elements suggested by the reviewer.

**Reviewer #2: General Comments**

• The paper will benefit from few typos and grammar errors e.g. “due inadequate preparation before its rollout...” We have now proof read the article and made all grammatical corrections.

• A section on limitation is missing. This should either come out clearly in the discussion section or have a stand-alone section. We have now made this adjustment and added the limitation section at the end of the discussion.

**Title**

• Very relevant and useful to literature and policy framing particularly in developing country settings. We thank the reviewer for this positive comment.

• However, the manuscript title is a little bit winding. I suggest that the title could just read “The role of actors in the policy formulation and implementation process: A policy of Kenya Free Maternal Health Policy.” We have now reworded the title to flow better

**Abstract**

• Loaded and not straight forward e.g.

“We conducted an exploratory qualitative study, which involved document review, key informant interviews (KIIs) with national stakeholders, and indepth interviews with County officials and health care workers (HCWS). We used a theoretical framework capturing the preliminary situation analysis of the policy, the processes, the content, and the stakeholders' roles in the formulation and implementation. This study was conducted in three facilities (levels 3, 4, and 5) in Kiambu County in Kenya.”

We have now rewritten the abstract to make it flow better and capture elements as suggested by reviewers.

• Three sentences as quoted above may be merged into two to read well and straightforward.

“Policy formulation or change requires the agents to work within the relevant context, stakeholder interests, power, ideas and framing of issues.”

We have now rewritten the abstract to make it flow better and capture elements as suggested by reviewers.

• This last statement in the abstract as quoted above serves no purpose. Is a statement of purpose, conclusion, or recommendation – for me the statement is not properly placed and should be taken out.

We have now rewritten the abstract to make it flow better and capture elements as suggested by reviewers.

**Introduction**

• Well written and captures salient aspects. We thank the reviewer for this positive comment.

• However, a few re-structuring will create a good flow. For example, line 108 to 116 states, “Kenya has made some good progress towards reducing neonatal and mortality rates and …. Kenya has made some good progress towards reducing neonatal and mortality rates and” (lines 108 -116). This entire paragraph may be captured in the opening paragraph to provide a good context of the “free” policy success to lay bare the rationale for examining its formulation and implementation. The usefulness of the policy gets buried where it is currently located. We thank the reviewer for this comment. We have now reformulated the introduction section as suggested by the reviewer to provide a better context.

**Methodology**

The guiding conceptual and analytical framework

• Well grounded. However, references mainly cited published articles.

We thank the reviewer for this positive comment.

Design

• Appropriate

We thank the reviewer for this positive comment.

**Setting**

“Kiambu County was chosen because of the logistic feasibility of data collection and the sociodemographic characteristics, health indicators and population size…”

• Logistical challenges are not good enough scientific reason for choosing a study site. This MUST always be accompanied by a compelling need for the site to be chosen. This is conspicuously mission in the section. Although, the authors cited sources to readers, authors have a duty to state what in those sources makes a compelling case for this county to be chosen. The population demographic stated afterwards don’t justify the choice either.

We thank the reviewer for this comment. Under the section on the study setting, we have now explained in detail the four main reasons why on the county section, Kiambu was selected from lines 285-305.

**Study population and selection.**

• Wide enough yet left out community opinion leaders and perhaps pregnant women.

“…county department of health officials; facility-in-charge and HCWs and others.” (Line 151 to 155).

• Who are the others in the above sentence? If these are traditional authorities or anyone from the community, it should stand out and as they are very critical in public policy formulation process. Otherwise, it should appear in the manuscript as a limitation of data gathering. You later read other in Table 5 to refer to Beyond Zero, Jacaranda Health etc and get a little bit confusion. Define others clearly and state whether or not traditional/opinion leaders were part of the process.

We thank the reviewer for this comment. In the section on the study population and sampling, we have detailed the study participants and their roles. The reviewers asked why we did not include community opinion leaders and pregnant women. We captured the perspectives of community opinion leaders through civil society and union representatives. While we acknowledge that including more perspectives would have been beneficial, we highlighted this as a limitation due to funding and timeline constraints. Although the opinions of mothers were not included in this study, they are thoroughly covered in other publications to avoid repetition. The concepts from mothers were used to complete the tables on the roles of stakeholders. Aligning with the reviewers' suggestions, we aimed to make the work concise and avoid redundancy. The information in Table 5 that refer to Beyond Zero, Jacaranda Health etc were gathered in details from the document reviews and we have now captured so clearly.

**Results**

**“Preliminary situation analysis, setting the priorities, and the context of the policy.”**

• The word “preliminary” is a little bit confusion and makes it look it the authors are presenting a ‘preliminary’ situational analysis rather than the first step of the policy formulation process. This is also reflected in the discussion (This preliminary situation analysis shows that the adoption /formulation of this policy followed the opening of a ‘window of opportunity”) section (line 656) and makes it worse. My considered opinion is that the authors adapt the theme to read without the word ‘preliminary’. It perhaps will read well and clear.

We thank the reviewer for this comment and suggestion. We have clearly defined the section on what we would like to present starting with the definition of the framework. In this part we have now showed that we are outlining the background priorities and context in which the policy was implemented.

• Critical quotes are mission from the opening results, yet the authors made references to same. “...for example, as noted by the respondents, the constitution provided every citizen with the right to quality health and life (including maternal and neonatal care)...” - Which respondent and where did they note it?

We thank the reviewer for this comment and suggestion. We have now corrected this section based on the fact that it came from document reviews.

• Solitary quotes referred to as bases for themes/subtheme e.g. The need to align with the global goals of achieving sustainable development goals (SDGs). The SDGs are a critical component of every country’s health systema and policy directive and as such should appear in not just from one KII. This should copiously be referred to in the results section. It never appears adequately in the interview responses then this should be highlighted in the discussion section. Other themes such “…to achieve political agenda…” is ok to have solitary quotes as this is a common knowledge in LMICs political space.

We thank the reviewer for this comment and suggestion. We have now corrected this section to show that they both came from the document reviews and the sentiments from the respondents. Further, throughout the whole document, we have reflected and shown its link to SDG.

**Discussion**

• “…Our findings show that the policy redesign envisaged expanding coverage and enhancing”.

• Why is the word policy redesign being introduced at this section? It is not clear to me.

We have now rewritten this section of the discussion section to mean expanded policy design.

• The actors leave out chiefs and community/opinion leaders who are rather critical in sub-Saharan Africa. Although, elected leaders are technically representatives of chiefs and opinion leaders, in policy formulation process are the FMS, their role is essential and should be stated as policy formulation process gap, if it was missing in the process. Alternatively, if the authors did not collect data on same, then this should be stated as limitation of data collection. We have included in the last paragraph of the discussion the limitation of the study for which this is part.

**Conclusion**

• The conclusion is rather a little off to me – the main aspects have it as,

“Our study highlights the basis for changing the previous policy to the current one driven by multiple agendas, including political determinants, the need to achieve global and national goals, and learnings from the previous policy. The interconnectedness of these drivers shaped the content and policy formulation processes”.

We have now rewritten the conclusion section and made it better flowing as suggested.

• I though the authors would rather conclude on bases of the framework upon which the study was grounded and state whether the process of formulation and implementation followed their chosen farmwork of study and why not.

We have now rewritten the conclusion section and made it better flowing as suggested.

• “Understanding policy processes is relevant across other LMICs, and we hope that this framework contributes to policy analysis and learning in Kenya and beyond”-

• This is similar to what is captured in the abstract ending. It serves little to no purpose to me. It can be taken out with losing anything relevant.

We have now rewritten the conclusion section and made it better flowing as suggested.
